# A scientometric dataset on pharmaceuticals and illicit drugs (including cocaine) in the water resources of Latin America and the Caribbean (1990–2024)

**DOI:** 10.1016/j.dib.2025.112100

**Published:** 2025-09-23

**Authors:** Vinicius Roveri, Alberto Teodorico Correia, Walber Toma, Aírton Zogaib Rodrigues, Camilo Dias Seabra Pereira, Luciana Lopes Guimarães

**Affiliations:** a Universidade Metropolitana de Santos (UNIMES), Avenida Conselheiro Nébias, 536 - Encruzilhada, 11045-002, Santos, São Paulo, Brasil; b Centro Interdisciplinar de Investigação Marinha e Ambiental (CIIMAR/CIMAR), Avenida General Norton de Matos S/N, 4450-208 Matosinhos, Portugal; c Laboratório de Pesquisa em Produtos Naturais, Universidade Santa Cecília (UNISANTA), Rua Cesário Mota 8, F83A, 11045-040 Santos, São Paulo, Brasil; d Instituto de Ciências Biomédicas Abel Salazar (ICBAS), Universidade do Porto (UP), Rua de Jorge Viterbo Ferreira 228, 4050-313 Porto, Portugal; e Departamento de Ciências do Mar, Universidade Federal de São Paulo (UNIFESP), Campus Baixada Santista, 11030-100 Santos, São Paulo, Brasil

**Keywords:** Scientometric mapping, SDG 06, Regional water pollution, Emerging pollutants, Ecological risk assessment, Research trends

## Abstract

This article presents a scientometric dataset on pharmaceuticals and illicit drugs in water resources across Latin America and the Caribbean (1990–2024). Bibliographic records were retrieved from the Web of Science™ Core Collection and processed using Bibliometrix (R environment), with metadata harmonised to ensure consistency. The dataset comprises ten figures and nine supplementary tables, covering annual publication trends, country productivity, co-authorship and keyword networks, and core journals. All resources are openly available via Mendeley Data (doi:10.17632/6y8pb89dnk.1), enabling direct access and reuse. This dataset provides a structured basis for analysing regional research capacity, collaboration patterns, and thematic hotspots. It can be integrated into comparative studies, systematic reviews, or machine learning approaches to identify knowledge gaps and support evidence-based policies on water quality and public health.

## Highlights

• First structured dataset on pharmaceuticals and illicit drugs in LAC waters.
• 154 WoSCC articles harmonised and curated with Bibliometrix (R).
• 10 figures and 9 tables via Mendeley Data (DOI: 10.17632/6y8pb89dnk.1).
• Enables trend analyses, systematic reviews, and policy-relevant insights.
• Supports comparative studies and machine-learning applications.

## Graphical abstract

**Figure:**
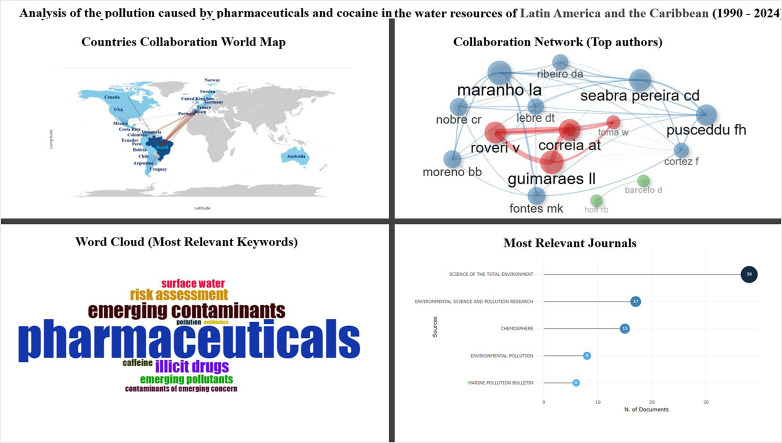

Specifications Table

**Table:** 

Subject	Earth & Environmental Sciences
Specific subject area	Scientometric dataset on pharmaceuticals and illicit drugs in Latin American and Caribbean (LAC) water resources
Type of data	Figures (.png); Tables (.xlsx). Processed and analysed data outputs from bibliometric software
Data collection	Records retrieved from the Web of Science™ Core Collection (WoSCC) database covering 1990–2024 (first record indexed in 2005). Data were exported in plain text format with full metadata (authors, affiliations, countries, keywords, citations, and sources). Bibliometric analyses were performed using Bibliometrix 4.4.3 within R 4.3.2, employing the Biblioshiny interface. Metadata harmonisation and cleaning were conducted within Bibliometrix functions, correcting duplicate author names, inconsistent institutional names, and keyword variations to ensure dataset consistency.
Data source location	Data source: WoSCC (Clarivate Analytics). Geographical focus: LAC: [WoSCC: https://clarivate.com/academia-government/scientific-and-academic-research/research-discovery-and-referencing/web-of-science/web-of-science-core-collection/]
Data accessibility	Repository name: Mendeley Data Data identification number (DOI): 10.17632/6y8pb89dnk.1 Direct URL to data: https://data.mendeley.com/datasets/6y8pb89dnk/1 Instructions for access: Openly accessible (public access via repository) [1].
Related research article	None.

## 1 Value of the Data

• Educational and methodological resource: The dataset provides a reproducible case study for training in bibliometric analysis and science mapping [2].
• Baseline dataset for scientometric research: These data provide the first structured bibliometric dataset on pharmaceuticals and illicit drugs (cocaine) in Latin American and Caribbean (LAC) water resources (1990–2024). Similar datasets have been successfully used in global assessments of pharmaceuticals and pollutants of emerging concern (PECs) [[3], [4], [5], [6], [7], [8], [9], [10], [11], [12]].
• Support for environmental monitoring and policy: Policymakers and environmental agencies may reuse these data to evaluate regional research capacity, identify knowledge gaps, and support strategies aligned with Sustainable Development Goals (SDG 6 and SDG 14) [[13], [14], [15], [16]].
• Identification of collaboration networks: By including author affiliations, co-authorship patterns, and country-level contributions, the dataset enables the mapping of international collaboration, following methodologies applied in previous scientometric reviews [17,18].
• Reuse in bibliometric and meta-analysis studies: The dataset can be integrated with other bibliographic repositories (e.g., Scopus, PubMed, Google Scholar) for comparative analyses and validation of scientometric methods [3,4,7].

## 2 Background

The dataset was compiled to provide a structured scientometric overview of research addressing the occurrence and ecological assessment of pharmaceuticals and illicit drugs in water resources across LAC. The motivation for generating this dataset arises from the increasing concern about PECs in aquatic environments [[5], [6], [7], [8], [9], [10], [11], [12], [19], [20]] and the absence of comprehensive bibliometric evaluations focusing on this region.

The methodological foundation of the dataset is scientometric analysis, which employs quantitative approaches to characterise the development of scientific output, research networks, and thematic hotspots [2,18]. Data were systematically retrieved from the Web of Science™ Core Collection (WoSCC) [3,4], processed using Biblioshiny (Bibliometrix 4.4.3, R environment), and curated to ensure transparency and reproducibility. Exclusion criteria were explicitly defined: only English-language peer-reviewed journal articles were included, while grey literature (e.g., theses, reports, and conference proceedings) was excluded.

## 3 Data Description

The dataset underpinning this article comprises two complementary components: (i) ten figures generated from scientometric and bibliometric analyses, and (ii) an Excel supplementary file containing both raw and processed data outputs, including tables and descriptive metadata. Together, these materials provide a comprehensive and structured overview of research addressing pharmaceuticals and cocaine in water resources across LAC.

## Figures

**Fig. 1:**
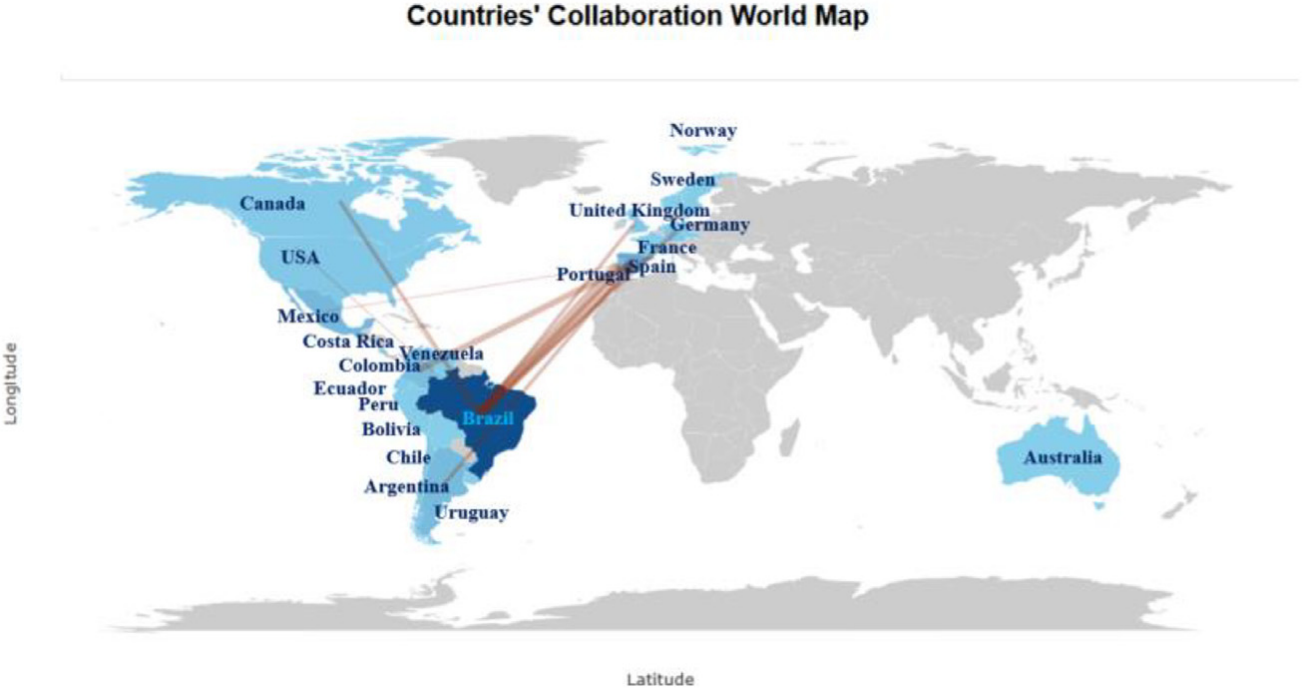
The map, created by Biblioshiny, shows cooperation between countries. The 11 countries within Latin America that had study records on the presence of pharmaceuticals and cocaine in Latin American and the Caribbean water resources were (in order of importance) Brazil, Mexico, Argentina, Costa Rica, Colombia, Chile, Ecuador, Peru, Venezuela, Bolivia and Uruguay. These countries also had international cooperation networks, mainly with the United States, Canada, Norway, Sweden, the United Kingdom, Germany, France, Spain, Portugal and Australia. Note: The darker the blue colour, the greater the number of publications registered in that country; Countries in grey indicate a lack of studies; The greater the intensity of the brown line, the greater the cooperation between countries.

**Fig. 2:**
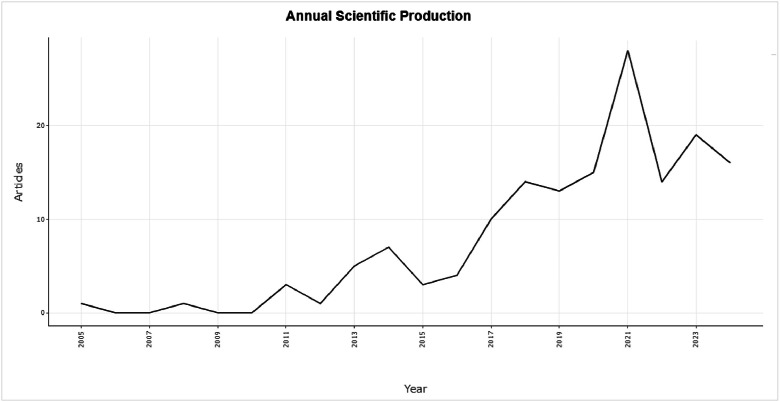
The graph, generated by Biblioshiny, shows the annual number of publications (range from 0 to 28 publications per year) indexed in Web of Science and published between 2005 and 2024.

**Fig. 3:**
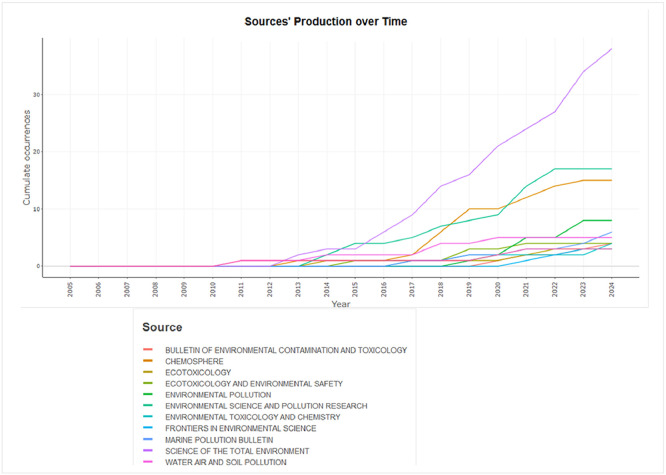
The graph, generated by Biblioshiny, shows the top 11 journals in terms of production over time, covering the period from 2005 to 2024. It is noteworthy that the journals Science of the Total Environment (Elsevier) and Environmental Science and Pollution Research (Springer Nature) account for about 33.3 % of all 154 publications.

**Fig. 4:**
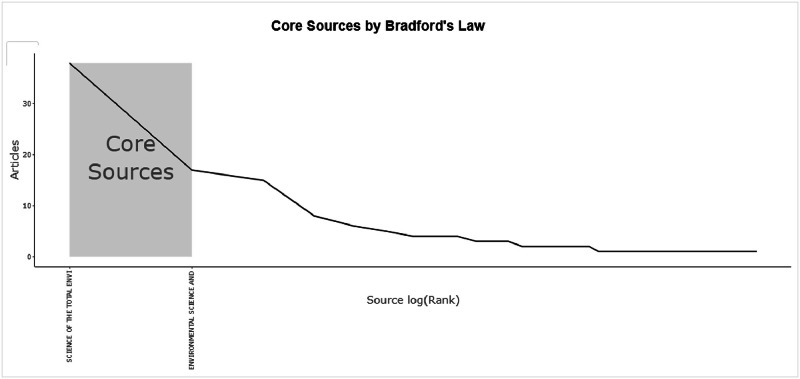
Core journals in accordance with Bradford's Law. The journals Science of the Total Environment (Elsevier) and Environmental Science and Pollution Research (Springer Nature) were discovered within zone 1, which comprises a small group of journals accounting for approximately 33.3 % of all publications (the Bradford's Law graph was generated by Biblioshiny).

**Fig. 5:**
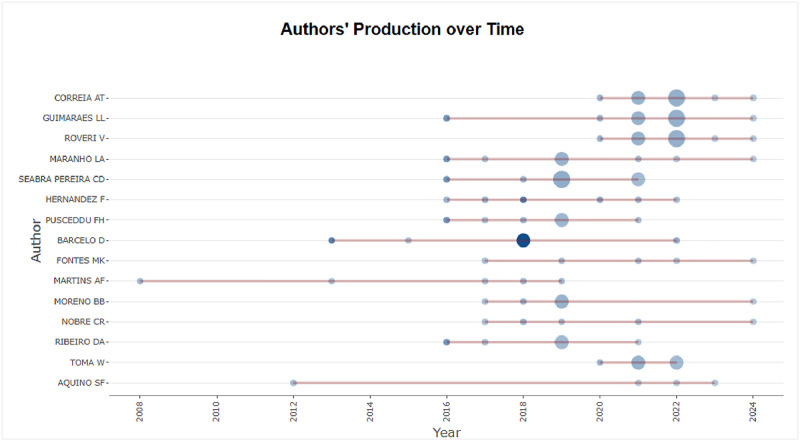
The graph generated by Biblioshiny shows the production of top authors over time. The period covered by the analysis is from 2008 to 2024. Note: The larger the number of circles, the greater the number of articles published by the author. The darker the circles, the greater the number of citations received.

**Fig. 6:**
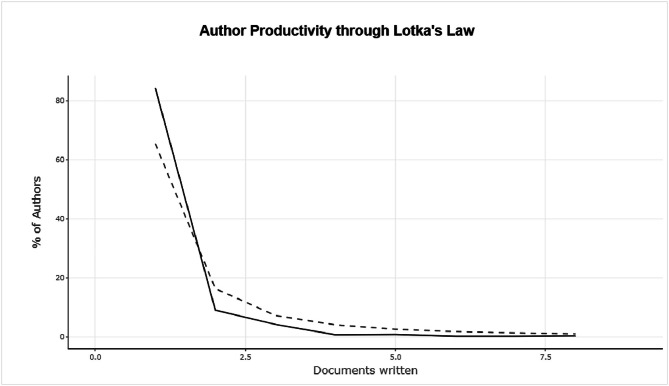
Lotka's Law deals with the frequency distribution of the productivity of authors in a given field. In this graph, generated by Biblioshiny, the frequency distribution categories are plotted: "X" is the number of papers and "Y" is the number of authors publishing x papers; the dashed line represents the theory of Lotka's law; the solid line represents the result of the study.

**Fig. 7:**
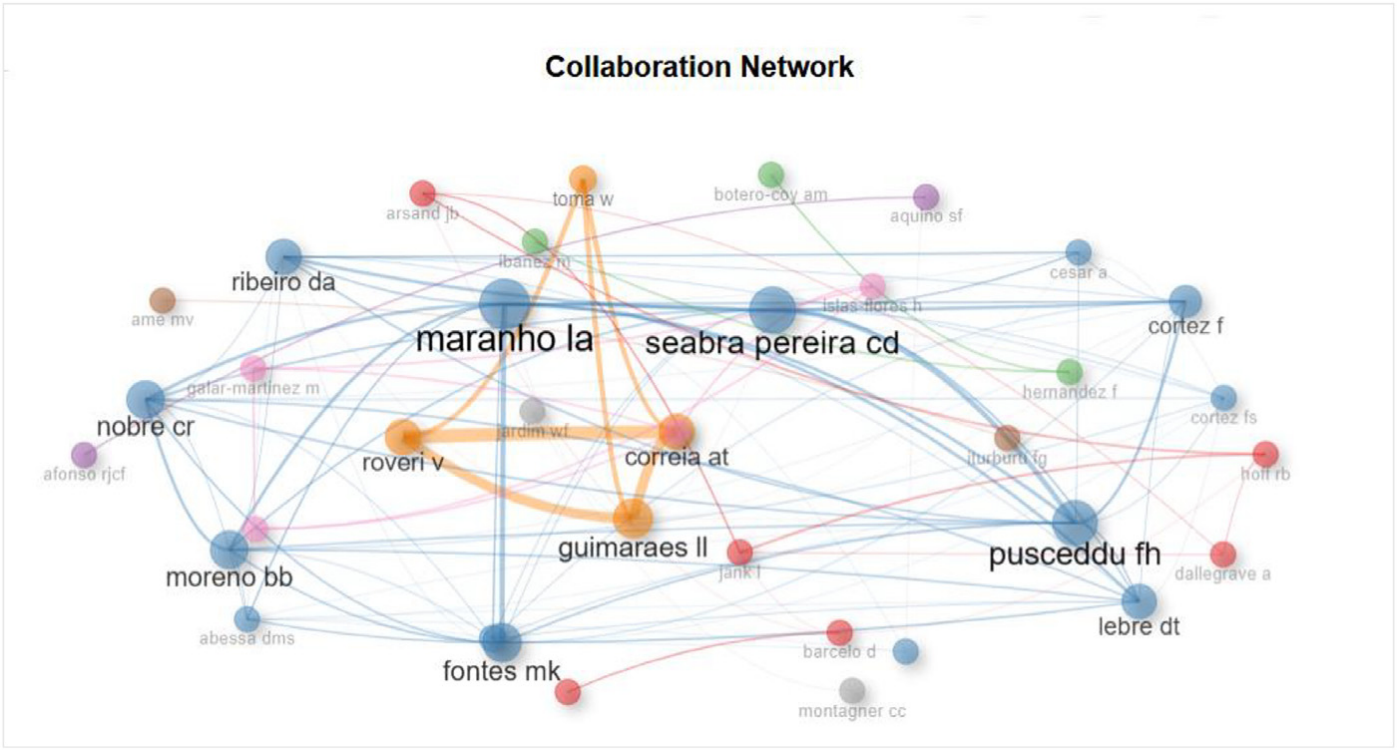
This network, generated by Biblioshiny, shows the results of the collaboration clusters of the main authors whose published articles deal with the occurrence of pharmaceuticals and cocaine in water resources in Latin American and the Caribbean. The analysis covers the period from 2005 to 2024. Researchers in the co-authorship network are linked by the number of publications they have co-authored. It is important to note that the size of each node indicates the relevance of the author (the larger, the more relevant), while the colour of each node indicates the clustering of the authors.

**Fig. 8:**
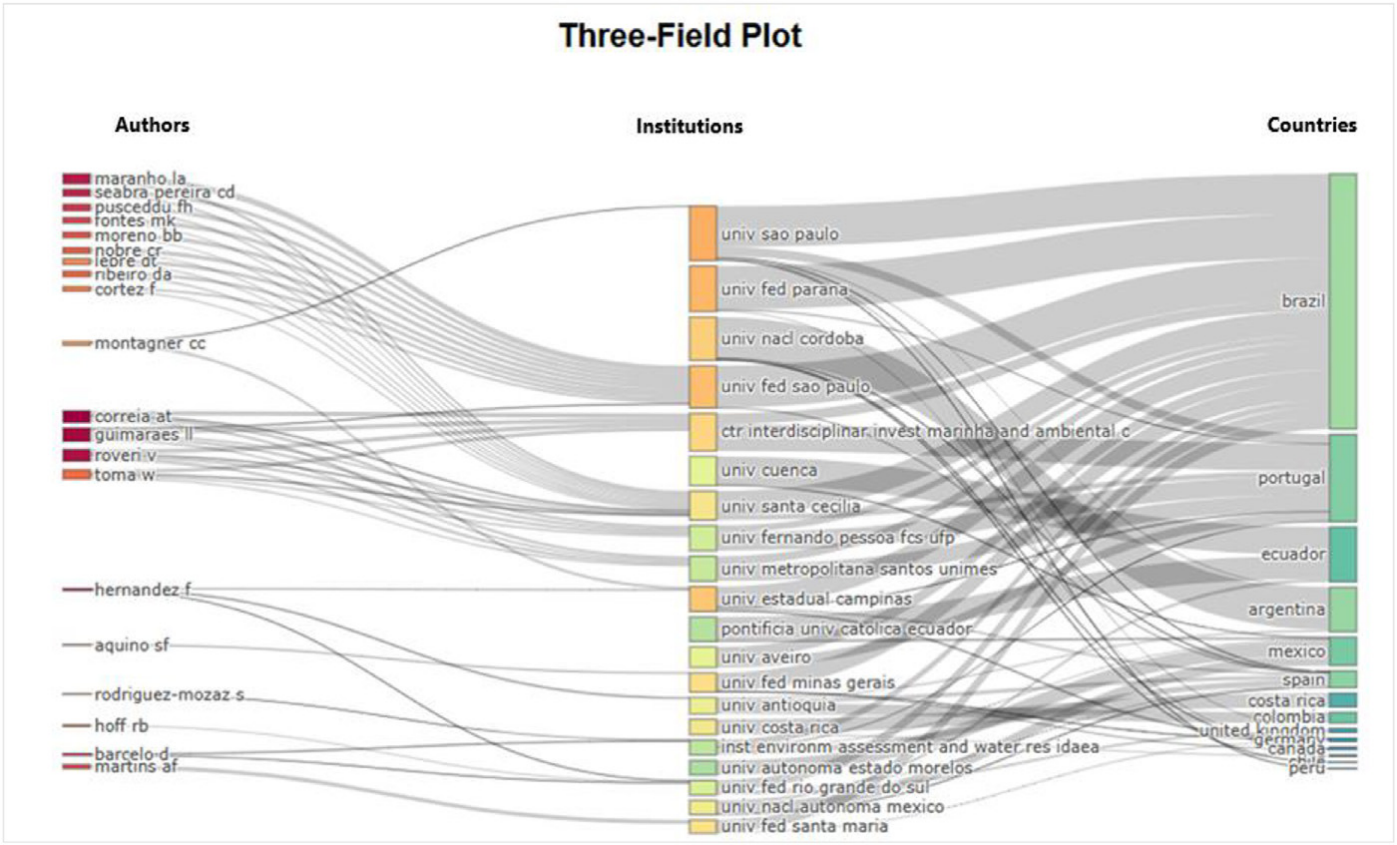
This three-field plot, generated by Biblioshiny, shows the relationships between authors (left), institutions (middle) and countries (right) resulting from the scientometric analysis of the pollution of Latin American and the Caribbean water resources by pharmaceuticals and cocaine (period 2005 to 2024).

**Fig. 9:**
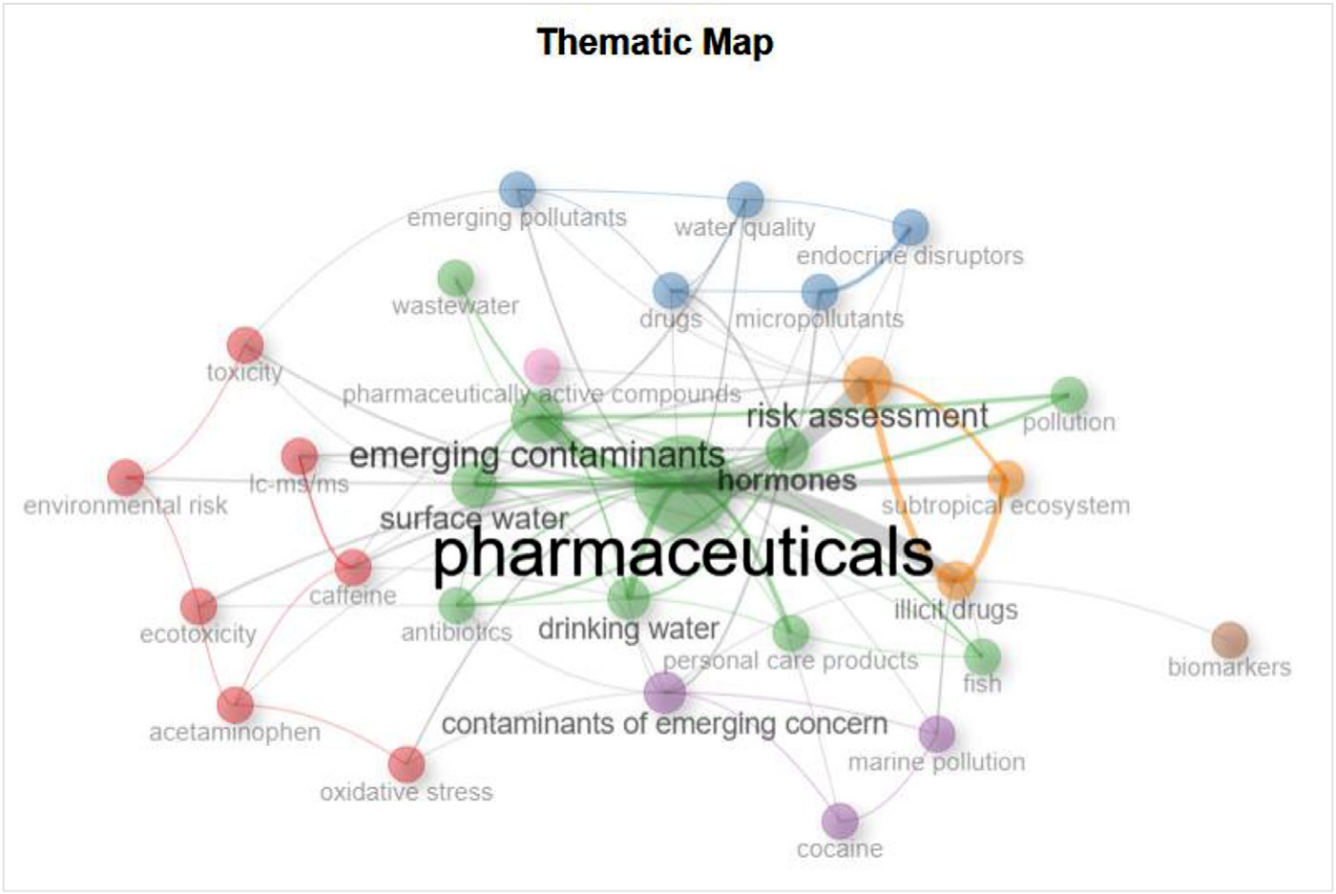
This network, generated by Biblioshiny, presents the results of keyword co-occurrence clusters from all published articles dealing with the occurrence of pharmaceuticals and cocaine in water resources in Latin American and the Caribbean. The period covered by the analysis is from 2005 to 2024. It is important to note that the size of each node indicates the importance of the keyword, while the colour of each node indicates the clustering of keywords.

**Fig. 10:**
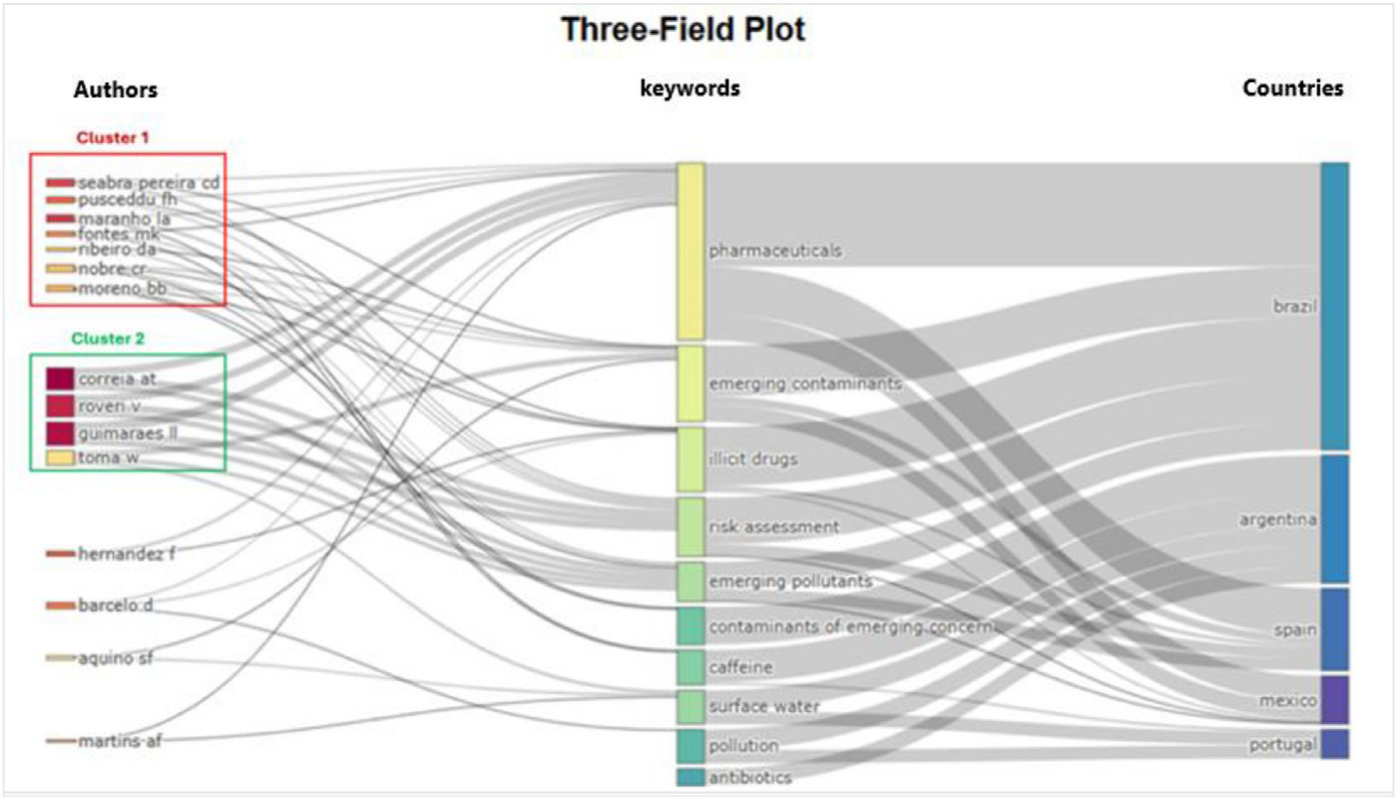
This three-field plot, generated by Biblioshiny, shows the relationships between authors (left), keywords (middle) and countries (right) resulting from the scientometric analysis of the pollution of Latin American and the Caribbean water resources by pharmaceuticals and cocaine (period 2005 to 2024).

The figures (Fig. 1, Fig. 2, Fig. 3, Fig. 4, Fig. 5, Fig. 6, Fig. 7, Fig. 8, Fig. 9, Fig. 10) provide visual representations of the scientometric outputs:

• Fig. 1. International cooperation map generated with Biblioshiny. It highlights the 11 Latin American countries with indexed publications on pharmaceuticals and cocaine in regional water resources (Brazil, Mexico, Argentina, Costa Rica, Colombia, Chile, Ecuador, Peru, Venezuela, Bolivia, and Uruguay). These countries established collaborative networks mainly with the United States, Canada, Norway, Sweden, the United Kingdom, Germany, France, Spain, Portugal, and Australia. Darker blue shades represent higher publication output, grey indicates no records, and the thickness of the brown lines reflects cooperation intensity.
• Fig. 2. Annual publication trends (2005–2024) retrieved from WoSCC and visualised in Biblioshiny. Yearly counts range from 0 to 28 publications, illustrating the growth of scientific production in this field, particularly after 2015.
• Fig. 3. Top 11 journals by publication output (2005–2024). Science of the Total Environment (Elsevier) and Environmental Science and Pollution Research (Springer Nature) are the most productive, together accounting for approximately 33.3 % of all 154 articles.
• Fig. 4. Core journals according to Bradford’s Law. Zone 1 includes Science of the Total Environment (Elsevier) and Environmental Science and Pollution Research (Springer Nature), which collectively represent ∼33.3 % of the publications and demonstrate their centrality in the field.
• Fig. 5. Temporal distribution of publications by the most prolific authors (2008–2024). Circle size reflects the number of publications, while darker shading indicates higher citation counts.
• Fig. 6. Lotka’s Law analysis of author productivity. The dashed line represents Lotka’s theoretical distribution, while the solid line corresponds to empirical results from the dataset.
• Fig. 7. Co-authorship network (2005–2024). Nodes represent researchers, with size indicating relevance (greater publication/citation counts) and colour denoting collaboration clusters. Links represent co-authorship intensity. The figure highlights the dominant role of Brazil in international research collaboration networks.
• Fig. 8. Three-field plot connecting authors (left), institutions (middle), and countries (right), based on scientometric data (2005–2024). The plot illustrates the relationships between researcher productivity, institutional contributions, and national affiliations.
• Fig. 9. Keyword co-occurrence network (2005–2024). Node size indicates keyword importance, while colour reflects thematic clusters. The network highlights emerging hotspots such as ‘emerging contaminants’ and ‘risk assessment.’
• Fig. 10. Three-field plot linking authors (left), keywords (middle), and countries (right) from 2005 to 2024, showing the association between leading researchers, research topics, and geographical scope.

## Supplementary Excel File

The supplementary dataset is organised into 9 sheets, corresponding to the 9 supplementary tables (S0–S8):

• Table S0 – Raw files (WoSCC): exported records (154 scientific articles).

• Table S1 – Annual Scientific Production: yearly publication counts.

• Table S2 – Sources’ Production over Time: journal output by year.

• Table S3 – Most Relevant Sources: ranking of contributing journals.

• Table S4 – Core Sources (Bradford’s Law): identification of zone-1 journals.

• Table S5 – Authors’ Production over Time: output by author across the study period.

• Table S6 – Lotka’s Law Productivity Distribution: theoretical vs. empirical productivity.

• Table S7 – Most Relevant Affiliations: institutions ranked by publication output.

• Table S8 – Completeness of Metadata: assessment of bibliographic data quality and consistency.

Note: The Excel file also includes two utility sheets (“General Information” and “Main Information”) that support navigation and descriptive statistics; they are not counted among the supplementary tables (S0–S8).

## 4 Experimental Design, Materials and Methods

Records were retrieved from the WoSCC database [2,3]. The timespan considered was 1990–2024, although the first indexed study was published in 2005 [[4], [5], [6], [7], [8]]. All records were exported in plain text format with full metadata (author names, institutional affiliations, countries, keywords, source titles, and citation information) [5,19,20].

Scientometric analyses and science mapping were performed using Bibliometrix 4.4.3 within the R environment (version 4.3.2). The Biblioshiny interface was employed to generate descriptive statistics and graphical outputs [2]. Data cleaning and harmonisation of author names, institutions, and keywords were performed to ensure metadata consistency. Duplicates and irrelevant records were excluded. Only peer-reviewed articles in English were retained, while grey literature (e.g., theses, reports, conference proceedings) was excluded [2,18].

The dataset is composed of two integrated components: Fig. 1, Fig. 2, Fig. 3, Fig. 4, Fig. 5, Fig. 6, Fig. 7, Fig. 8, Fig. 9, Fig. 10.

These include graphical representations generated through Bibliometrix/Biblioshiny and further refined for visual clarity. Figures illustrate research trends, collaboration networks, journal productivity, authorship distribution, keyword co-occurrence, and institutional contributions.

Supplementary Tables (S0–S8).

Data tables were extracted from bibliometric outputs and organised in Microsoft Excel (.xlsx). Each table corresponds to a specific dimension of the bibliometric analysis:

○ Table S0: Raw files (WoSCC)
○ Table S1: Annual scientific production
○ Table S2: Sources' Production over Time
○ Table S3: Most relevant sources
○ Table S4: Core sources (Bradford’s Law)
○ Table S5: Authors’ production over time
○ Table S6: Lotka’s Law productivity distribution
○ Table S7: Most relevant affiliations
○ Table S8: Completeness of metadata

Roadmap of Dataset Components

**Table:** 

Item	Description	Function
Fig. 1	Map of international cooperation among countries	Visualises research collaboration networks
Fig. 2	Annual publication trends (2005–2024)	Depicts yearly growth in scientific output
Fig. 3	Most productive journals	Identifies journals with the highest output
Fig. 4	Core journals (Bradford’s Law)	Defines core journals by citation distribution
Fig. 5	Most prolific authors	Ranks leading contributors in the field
Fig. 6	Lotka’s Law of author productivity	Compares theoretical and empirical productivity
Fig. 7	Co-authorship network	Illustrates patterns of researcher collaboration
Fig. 8	Three-field plot (authors–institutions–countries)	Maps interconnections between authors, affiliations, and countries
Fig. 9	Keyword co-occurrence network	Highlights thematic clusters and research hotspots
Fig. 10	Three-field plot (authors–keywords–countries)	Shows relationships between researchers, keywords, and geographical scope
Table S0	Raw files (WoSCC)	Exported records (154 scientific articles)
Table S1	Annual scientific production	Yearly number of publications retrieved
Table S2	Sources' Production over Time	Yearly number of productions over Time
Table S3	Most relevant sources	Ranking of journals publishing in the field
Table S4	Core sources (Bradford’s Law)	Identification of core journals (zone 1)
Table S5	Authors’ production over time	Output of individual authors across the study period
Table S6	Lotka’s Law productivity distribution	Statistical validation of author productivity
Table S7	Most relevant affiliations	Institutional contribution ranked by publication output
Table S8	Completeness of metadata	Assessment of bibliographic data quality and consistency

The combined dataset provides a structured bibliometric overview of the occurrence and study of pharmaceutical and cocaine compounds in water resources across LAC.

## Limitations

This dataset has some limitations:

• Coverage is restricted to the Web of Science™ Core Collection (WoSCC); relevant studies from Scopus, PubMed, or regional databases may be missing.
• Only English-language peer-reviewed journal articles were included. Spanish and Portuguese publications were excluded, which may underrepresent regional literature.
• Grey literature (e.g., theses, technical reports, and conference proceedings) was excluded, which can limit policy comprehensiveness.
• Records are current up to 31 December 2024; studies published after this date are not included.
• Some WoSCC records contained incomplete author affiliation data, which may affect collaboration-network analyses.
  Despite these constraints, this dataset provides a reproducible, structured foundation for scientometric and bibliometric research.

## Ethics statement

Not applicable. This dataset was generated entirely from bibliographic records retrieved from the Web of Science® Core Collection (Clarivate Analytics). No experiments involving humans, animals, or sensitive data were conducted.

## CRediT Author Statement

**Vinicius Roveri:** Conceptualization; Data curation; Methodology; Formal analysis; Writing – original draft; **Alberto Teodorico Correia:** Formal analysis; Validation; Writing – review & editing; **Walber Toma:** Formal analysis; Data interpretation; Writing – review & editing; **Aírton Zogaib Rodrigues:** Formal analysis; Writing – review & editing; **Camilo Dias Seabra Pereira:** Formal analysis; Writing – review & editing; **Luciana Lopes Guimarães:** Formal analysis; Writing – review & editing.

## Funding

Alberto Teodorico Correia was supported by National funds through the Foundation for Science and Technology (FCT), under projects UIDB/04423/2020 and UIDP/04423/2020.

C.D.S. Pereira acknowledges the support of the 10.13039/501100003593National Council for Scientific and Technological Development (CNPq, Brazil) through the Universal Project (grant #406013/2025-0) and a Research Productivity Fellowship (grant #308408/2022-5).

## Data Availability

Mendeley DataScientometric dataset on pharmaceuticals and illicit drugs in Latin American and Caribbean water resources (Original data).

## Appendix Supplementary materials

Image, application 1
